# Improved Outcomes with Intensity Modulated Radiation Therapy Combined with Temozolomide for Newly Diagnosed Glioblastoma Multiforme

**DOI:** 10.1155/2014/945620

**Published:** 2014-01-19

**Authors:** Noel J. Aherne, Linus C. Benjamin, Patrick J. Horsley, Thomaz Silva, Shea Wilcox, Julan Amalaseelan, Patrick Dwyer, Abdul M. R. Tahir, Jacques Hill, Andrew Last, Carmen Hansen, Craig S. McLachlan, Yvonne L. Lee, Michael J. McKay, Thomas P. Shakespeare

**Affiliations:** ^1^Department of Radiation Oncology, North Coast Cancer Institute, Coffs Harbour, NSW, Australia; ^2^Rural Clinical School Faculty of Medicine, University of New South Wales, NSW, Australia; ^3^Department of Radiation Oncology, North Coast Cancer Institute, Lismore, NSW, Australia; ^4^Department of Radiation Oncology, North Coast Cancer Institute, Port Macquarie, NSW, Australia; ^5^Faculty of Medicine, University of Sydney, NSW, Australia

## Abstract

*Purpose*. Glioblastoma multiforme (GBM) is optimally treated by maximal debulking followed by combined chemoradiation. Intensity modulated radiation therapy (IMRT) is gaining widespread acceptance in other tumour sites, although evidence to support its use over three-dimensional conformal radiation therapy (3DCRT) in the treatment of gliomas is currently lacking. We examined the survival outcomes for patients with GBM treated with IMRT and Temozolomide. *Methods and Materials*. In all, 31 patients with GBM were treated with IMRT and 23 of these received chemoradiation with Temozolomide. We correlated survival outcomes with patient functional status, extent of surgery, radiation dose, and use of chemotherapy. *Results*. Median survival for all patients was 11.3 months, with a median survival of 7.2 months for patients receiving 40.05 Gray (Gy) and a median survival of 17.4 months for patients receiving 60 Gy. *Conclusions*. We report one of the few series of IMRT in patients with GBM. In our group, median survival for those receiving 60 Gy with Temozolomide compared favourably to the combined therapy arm of the largest randomised trial of chemoradiation versus radiation to date (17.4 months versus 14.6 months). We propose that IMRT should be considered as an alternative to 3DCRT for patients with GBM.

## 1. Introduction

Gliomas are the most common primary brain tumour, and GBM accounts for up to 70% of cases. The prognosis is poor, and while adjuvant cranial irradiation has been shown historically to significantly improve survival rates [1, 2], the treatment of patients with GBM remains challenging. The median survival for patients with GBM treated with postresection radiation alone has been of the order of 11 months. Recent advances in chemotherapy have increased overall survival to around 14.6 months with 26% survival at 2 years with the addition of concurrent and adjuvant Temozolomide [3]. This improvement in survival is even greater for those patients with favourable molecular profiles. In the phase 3 trial from the European Organisation for Research and Treatment of Cancer published by Hegi et al., there was a 46% 2 year survival for those patients who had epigenetic silencing via methylation of the promoter of the gene which metabolises Temozolomide (O-6-methylguanine-DNA-methyltransferase, MGMT) [4]. This survival benefit was maintained on long-term followup [5]. While these advances in systemic treatment, cancer genomics, and the availability of highly conformal treatments such as IMRT are encouraging, they have not fully translated into clinical practice for treatment of these challenging tumours. While there have been a small number of contemporary series employing conventionally fractionated IMRT published to date [6, 7], the dosimetric advantages of IMRT have not translated into an improvement in reported survival. However, the presence of a small number of long-term survivors, the resultant increase in clinically evident radiation-related late neurocognitive effects [8], and the promising utility of IMRT at other treatment sites justify further investigation in the management of glioblastoma multiforme. This retrospective study from our centre reports a consecutive contemporary series of patients treated with either conventionally fractionated IMRT or hypofractionated IMRT for those with poor performance status. The majority of these patients also received Temozolomide chemotherapy.

## 2. Methods

Following institutional review board approval, the electronic medical records of eligible patients with histologically proven GBM who were treated with IMRT were reviewed.

### 2.1. Radiation Therapy Target Definition

All patients underwent computed tomographic (CT) simulation and were immobilised using a commercially available thermoplastic mask system. CT image data was reconstructed in 2 mm slice thickness and then coregistered with preoperative and postoperative magnetic resonance imaging (MRI). All MRI sequences were acquired pre- and postintravenous gadolinium contrast. Treatment volumes were as per our institutional protocol based on the Australian Cancer Network clinical practice guidelines for the management of adult gliomas [9]. The gross tumour volume (GTV) was defined as the enhancing lesion on T1 postgadolinium and T2 FLAIR MRI sequences. The clinical target volume (CTV) included the GTV with a 2 centimetre expansion, constrained by anatomical boundaries. Planning target volume was delineated as the CTV plus a concentric expansion of 5 mm. All patients were treated to either 40.05 Gy or 60 Gy prescribed to the International Commission of Radiological Units and Measurements reference point using a single phase IMRT technique.

### 2.2. Radiation Therapy Treatment Planning and Dose Prescription

All patients underwent inverse planning on the XiO treatment planning system (Elekta, Crawley, UK) and patients were treated with a five-field technique. The median prescribed dose for all patients was 60 Gy (range 40.05 Gy to 60 Gy).

### 2.3. Chemotherapy

All patients who received concurrent Temozolomide received 75 mg/m^2^ daily during IMRT and received Temozolomide 150 mg/m^2^ for five days in each 28-day cycle for six cycles following completion of IMRT with doses increasing to 200 mg/m^2^ as tolerated.

### 2.4. Toxicity Assessment

Baseline toxicity prior to initiation of IMRT and on treatment toxicity was available for all patients. Posttreatment toxicity data was available for 16 of 31 treated patients (51.6%). Toxicity was prospectively assessed according to the Common Terminology Criteria for Adverse Events (CTCAE) v3.0 and later v4.0. No patient had Grade 4 or Grade 5 toxicity at any time, and the most commonly reported events were Grade 2 cognitive disturbance, Grade 2 concentration impairment, or Grade 1 headache. No patient required amendment of their treatment plan due to any acute toxicity which could be ascribed to their treatment.

### 2.5. Patient Followup

All patients had baseline toxicity assessment prior to initiation of IMRT and were seen weekly with weekly toxicity assessment on treatment. All patients had posttreatment followup and toxicity assessment at one month and three months. Sequential MRI scans were undertaken three monthly to assess for radiological progression. Patients were offered reoperation or retreatment with Temozolomide or enrollment in a clinical trial on progression. Of 20 patients who had progressed at the time of analysis, 2 patients had reoperation alone, 1 patient had reoperation followed by Bevacizumab therapy, and 1 patient was treated with Carboplatin combined with Bevacizumab. The remaining patients died of disease without further intervention and no patient underwent reirradiation in this series.

### 2.6. Data Analysis

We performed descriptive analyses. Progression-free survival was measured from the date of diagnosis to the first clinically evident date of radiologically proven progression or date of death in those patients who had not been diagnosed with progression of disease. Overall survival was measured from the date of diagnosis to the date of death. No patient has been lost to followup. Survival curves were generated according to the Kaplan Meier methods and compared using the log rank test. All analyses were conducted using the Stata 11 statistical software package (Statcorp, College Station, TX, USA).

## 3. Results

### 3.1. Patient Characteristics

From April 2007 to June 2012, there were 31 patients with histologically proven GBM who underwent IMRT at the North Coast Cancer Institute. All patients had prior neurosurgery, of whom 3 patients (9.7%) had biopsy only, 9 patients (29%) had subtotal tumour resection, and 18 patients (58.1%) had gross total tumour resection. The extent of surgery could not be determined for 1 patient (3.2%). Of the 31 patients, 19 patients (61.3%) received 60 Gy at daily doses of 2 Gy and all but one of these patients received Temozolomide as per the current standard of care [3]. The remaining 12 patients (38.7%) received 40.05 Gy at daily doses of 2.67 Gy, because of poor performance status (Eastern Cooperative Oncology Group score ≥ 2) or patient preference. Of the 12 patients who received hypofractionated treatment, 6 patients were enrolled on a clinical trial which randomised elderly patients to Temozolomide and hypofractionated radiation versus hypofractionated radiation alone [10], of whom 5 patients received Temozolomide. The remaining 6 patients who received 40.05 Gy did not receive Temozolomide. Patient demographics are in Table 1.

**Figure 1:**
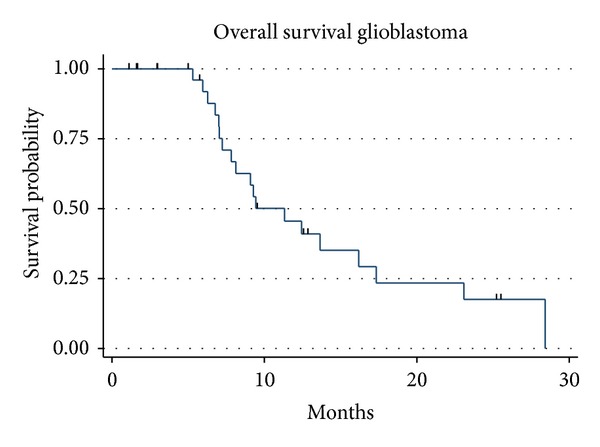
Kaplan Meier overall survival for all 31 patients for hypofractionated (blue line) and conventionally fractionated (red line) treatment.

**Figure 2:**
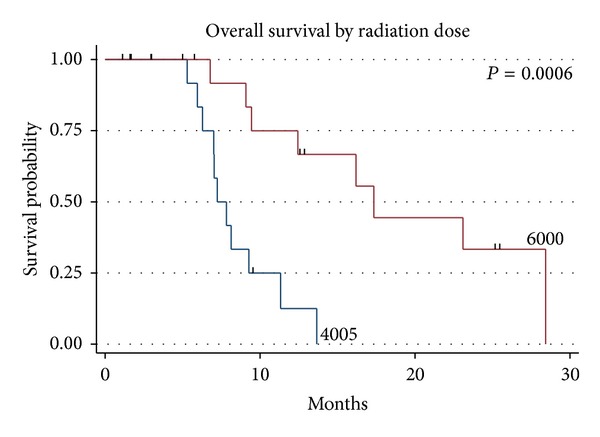
Kaplan Meier overall survival for all 31 patients for hypofractionated 4005 centiGray IMRT (blue line) and conventionally fractionated 6000 centiGray IMRT (red line).

**Figure 3:**
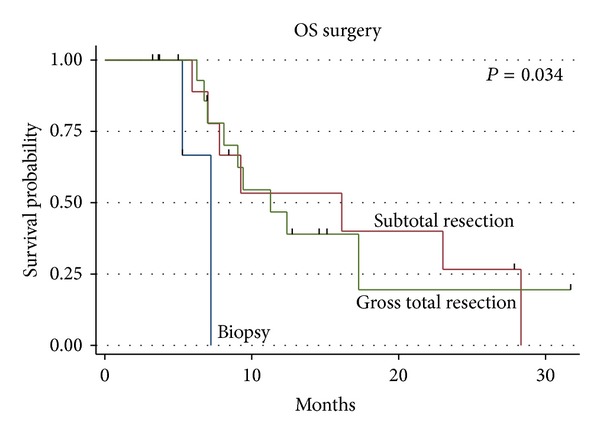
Overall survival by extent of surgical debulking.

**Figure 4:**
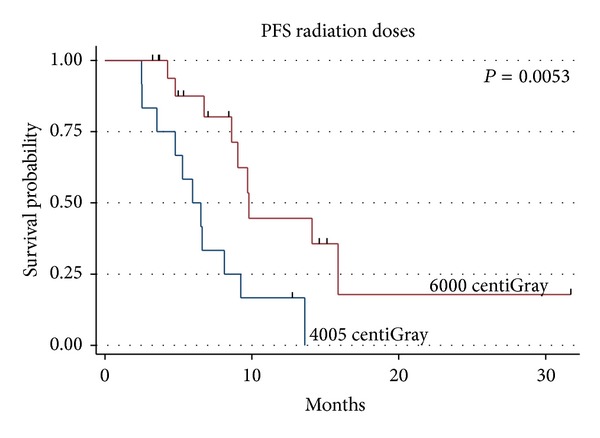
Progression-free survival by radiation dose.

Of the 31 patients, 19 (61.3%) were deceased at the time of last followup and 1 patient was alive with disease, with a median follow-up time of 9.4 months (range 3.2–31.6 months). The median survival was 11.3 months for the entire group (Figure 1). The median survival was 17.4 months for those receiving 60 Gy and 7.3 months for those receiving 40.05 Gy (*P* = 0.0006, log rank and Wilcoxon analysis, Figure 2), which is likely due to the more favourable patient characteristics of the group selected to receive 60 Gy. The difference in overall survival according to extent of surgical resection was also statistically significant (*P* = 0.034, Figure 3). A median Progression-free survival of 9.1 months was observed for the entire group, and the difference in Progression-free survival between those receiving 40.05 Gy and those receiving 60 Gy was statistically significant (*P* = 0.005, Figure 4).

## 4. Discussion

To our knowledge, the current series represents one of the largest published series of patients with primary GBM treated with IMRT combined with Temozolomide chemotherapy in the modern literature. Our series employed a standardised IMRT technique and Temozolomide chemotherapy schedule, and those patients receiving 60 Gray had a median overall survival of 17.4 months. While this is a retrospective series subject to the usual biases, this still compares favourably to previously published series of three-dimensional conformal radiation and Temozolomide [3], irrespective of MGMT methylation status as this was not available for our patients. In the paper by Stupp and colleagues [3], median overall survival was 14.6 months for those patients on the combined radiation therapy and Temozolomide arm, independent of MGMT methylation status.

The multimodality care of patients with glioblastoma multiforme has been evolving for the last several decades, but prognosis remains poor. Regarding systemic therapy, the use of concurrent and adjuvant Temozolomide combined with cranial irradiation compared to irradiation alone [3] has been shown to significantly improve overall survival. Advances in the field of radiation therapy have been the addition of cranial irradiation to debulking surgery alone [1], the establishment of a dose response relationship [11], and the use of altered fractionation schedules. These altered fractionation schedules have included abbreviated hypofractionated schedules in those patients with GBM with poor performance status [12] and dose escalation strategies, with the latter producing mixed results [13, 14]. Piroth et al. [13] employed an integrated boost IMRT technique in which 72 Gy at 2.4 Gy per fraction was prescribed to a clinical target volume derived from positron emission tomography scans. Median overall and Progression-free survival were 14.8 months and 7.8 months, respectively. In a Radiation Therapy Oncology Group phase one study, Tsien et al. [14] employed a sequential conformal boost technique to evaluate four dose levels; 66 Gy, 72 Gy, 78 Gy, and 84 Gy. Median overall survival at 19.3 months was highest for those patients receiving 84 Gy with planning target volumes less than 75 cm^3^.

A number of studies have been published which investigated the use of conventionally fractionated IMRT but in contrast to the current series described heterogenous populations. Fuller et al. [6] reported the use of tomotherapy, either for the entire treatment course or as a boost following three-dimensional conformal radiation therapy. However, a number of patients were treated for recurrent disease, and the use of chemotherapy was variable. Of a total of 42 patients described, only 7 patients received IMRT with Temozolomide. Fuller et al. reported a median overall survival of 8.7 months and cautioned against the use of IMRT in this population. Narayana et al. [7] reported a series from Memorial Sloan Kettering Cancer Centre of 58 patients with high grade gliomas which included 41 patients treated for glioblastoma multiforme. In that series, one-quarter of patients had biopsy only and 80% of patients received adjuvant or concurrent chemotherapy. It is not clear what proportion of these patients had glioblastoma multiforme and the type of chemotherapy was not specified. The median Progression-free survival and median overall survival in their study was 2.5 months and 9 months, respectively. Dosimetric comparison of IMRT and 3DCRT plans showed that IMRT did not yield clinically significant advantages in terms of planning target volume coverage. This might be expected, as contemporary conformal techniques give good target coverage, irrespective of intracranial tumour location. IMRT did offer an advantage in terms of reducing dose to critical normal structures and reduced dose to normal brain. This reduction in dose to critical normal structures as well as a reduction in integral dose to normal brain tissue has also been replicated in planning studies comparing IMRT to 3DCRT [15]. This has also been demonstrated in studies of IMRT at other disease sites, including head and neck cancer [16] and prostate cancer [17].

## 5. Conclusion

The outlook for patients with glioblastoma multiforme is improving incrementally, in part due to an increasing armamentarium of biologically active drugs, which include integrin inhibitors [18] and antiangiogenic agents [19]. These developments, in combination with greater knowledge of glioblastoma genomics and proteomics [20], better neuroimaging modalities including functional imaging [21], more targeted surgical techniques [22], and evolving radiation therapy technology, are increasing the proportion of patients, however small, that are becoming long-term survivors. As in our study, the combination of IMRT at standard radiation doses with Temozolomide can lead to an increase in median overall survival. It is likely that the biggest contribution of IMRT will be for those long-term survivors in the prevention of long-term neurocognitive morbidity which may be achieved by a reduction in dose to critical normal structures and normal brain tissue, even while escalating dose to the planning target volume. We conclude that IMRT may thus facilitate a therapeutic gain for patients with glioblastoma multiforme.

## Figures and Tables

**Table 1:** Patient demographics.

	(Percentage)	
Age (years)		
Median	63.9	
Range	30.7–89.2	
Gender		
Male	21 (67.7)	
Female	10	

	40.05 Gray	60 Gray

Functional status		
ECOG 0	2 (16.67%)	7 (36.9%)
ECOG 1	8 (66.66%)	12 (63.1%)
ECOG 2	2 (16.67%)	0

Surgery		
Biopsy/unknown	4 (12.9)	
Subtotal resection	9 (29)	
Gross total resection	18 (58.1)	

	Concurrent TMZ	Adjuvant TMZ

60 Gray (*n* = 19)	18	18
40.05 Gray (*n* = 12)	5	5
